# Enhancing the stability and catalytic efficiency of alkyl halide dehalogenase through poloxamer temperature-sensitive gel

**DOI:** 10.1371/journal.pone.0319810

**Published:** 2025-03-21

**Authors:** Jianjun Zhu, Jun Zhang, Feng Gong, Qiong Yu, Xuan Guo, Yu Wang

**Affiliations:** 1 State Key Laboratory of NBC Protection for Civilian, Research Institute of Chemical Defense, Academy of Military Science, Beijing, China; 2 Department of Stem Cell and Regenerative Medicine Laboratory, Institute of Health Service and Transfusion Medicine, Beijing, China; 3 The General Hospital of Western Theater Command, Chengdu, China; 4 Department of Laboratory Medicine, the First Medical Center, Chinese PLA General Hospital, Beijing, China; TARO Pharmaceuticals, CANADA

## Abstract

Dha A, a biocatalyst with pronounced efficacy in the degradation of mustard gas, is constrained by its inherent instability, which impedes its broader application. In this study, we encapsulated Dha A within a poloxamer-based thermosensitive hydrogel, a widely utilized protein carrier, to assess its physicochemical characteristics, catalytic performance, and stability enhancement. The Dha A-loaded thermosensitive gel (Dha A@TSG) exhibited interactions between Dha A and poloxamer molecules via hydrogen bonding, with an optimal gelation temperature of 25°C. This encapsulation strategy significantly enhanced the solubility and catalytic efficiency of the mustard gas mimic, bis(2-chloroethyl) ether, surpassing the performance of the free Dha A solution. At 32°C, the poloxamer molecules within Dha A@TSG formed a tightly packed stereostucture, which substantially improved the storage and thermal stability of Dha A. Collectively, our findings offer valuable technical insights into the stabilization and catalytic efficiency enhancement of Dha A through the employment of poloxamer thermosensitive gels.

## 1. Introduction

Mustard gas, as a potent vesicant chemical warfare agent, poses a great danger to human skin. The mustard gas contamination of human skin causes acute and chronic skin damages, such as erythema, blisters, ulceration, skin pigmentation, and itching. The current treatment strategies for these skin problems are infection prevention and wound healing [1–6]. Hence, it is important to develop a highly efficient and biocompatible skin decontaminant for mustard gas to rapidly degrade it and protect skin wounds at the early stage of contamination.

Biological enzymes are less corrosive and environment-friendly; thus, they are desirable decontaminants for human skin. Dha A (obtained from Rhodococcus rhodochrous) is a haloalkane dehalogenase known for its high catalytic efficiency [7–9]. Dha A can catalyze hydrolytic cleavages of carbon-halogen bonds in halogenated compounds to produce alcohols, hydrogen ions, and halides [10,11]. Dha A is an effective and mild catalyst for the biodegradation of halogenated compounds and the bioremediation of contaminated environments [12–17]. Although Dha A is an excellent biological enzyme with high catalytic degradation activity against mustard gas, poor stability limits its applications [18].

Immobilization can enhance the structural rigidity of an enzyme by exerting forces on it and building a supportive microenvironment. Immobilization methods include physical adsorption, encapsulation, covalent binding, crosslink and conjugation with polymers [19,20].For example, physical adsorption is one of the most widely used methods in enzyme immobilization because of its straightforward experimental procedure and the use of nontoxic solvents [21]. Zheng synthesized a sandwich-like structure of mesoporous foam modified with polyethylene glycol (PEG) and amino groups to immobilize Dha A [22]. Wang covalently combined arabinogalactan with Dha A for enzyme immobilization and noticed significant improvements in the storage stability, high salt concentration stability, pH stability, and thermal stability of the enzyme [9].

Carriers, as binding objects for biological enzymes, can influence the catalytic performance of enzymes, substrates, and reaction mediums during catalytic reactions. Temperature-sensitive hydrogels can exist in a liquid state at room temperature or at a specific temperature and undergo an immediate phase transition to a semi-solid gel state upon contact with human skin or at a specific temperature [15,23–26]. Poloxamer is a nonionic surfactant consisting of hydrophilic polypropylene oxide (PEO) and lipophilic polyethylene oxide (PPO) groups in ABA-type terpolymers. The type of poloxamer can be changed by altering the ratio of PPO and PEO [27–31]. Poloxamer is non-toxic, non-irritating, and biocompatible, and it gets metabolized by the liver and kidneys; thus, it has been used as a food additive and pharmaceutical excipient for a long time. Poloxamer can be solubilized in different types of formulations, enabling its use in multiple applications, such as for rectal, inhalation, ophthalmic, and topical preparations [32–34]. It is reported that temperature-sensitive gels loaded with proteins and peptide drugs can prolong the stability of loaded drugs [35,36].

In the present work, Dha A was immobilized by a poloxamer temperature-sensitive gel. In addition, the physicochemical properties of the gel were characterized. Furthermore, the effects of the temperature-sensitive gel on the catalytic performance and stabilization of Dha A were analyzed.

## 2. Results

### 2.1. Determination of gelation temperature and gelation time

The gelation temperature and gelation time of the Dha A@TSG were 25.2°C and 125 s, respectively, while the corresponding values for the blank temperature-sensitive gel were 25.4°C and 123 s, respectively (Table 1). Hence, it was clear that the addition of Dha A to the temperature-sensitive gel had no significant effect on the gelation temperature and gelation time of the gel.

**Table 1:** Gelation temperature and gelation time of the Dha A@TSG and the blank temperature-sensitive gel.

Gel name	Gelation temperature (°C)				Gelation time (s)			
	1	2	3	Mean ± SD	1	2	3	Mean ± SD
Dha A@TSG	25.1	25.2	25.4	25.2 ± 0.2	125	123	124	125 ± 1
Blank gel	25.3	25.6	25.3	25.4 ± 0.2	126	130	123	123 ± 3

### 2.2. Fourier-transform infrared spectroscopy

**Fig 1:**
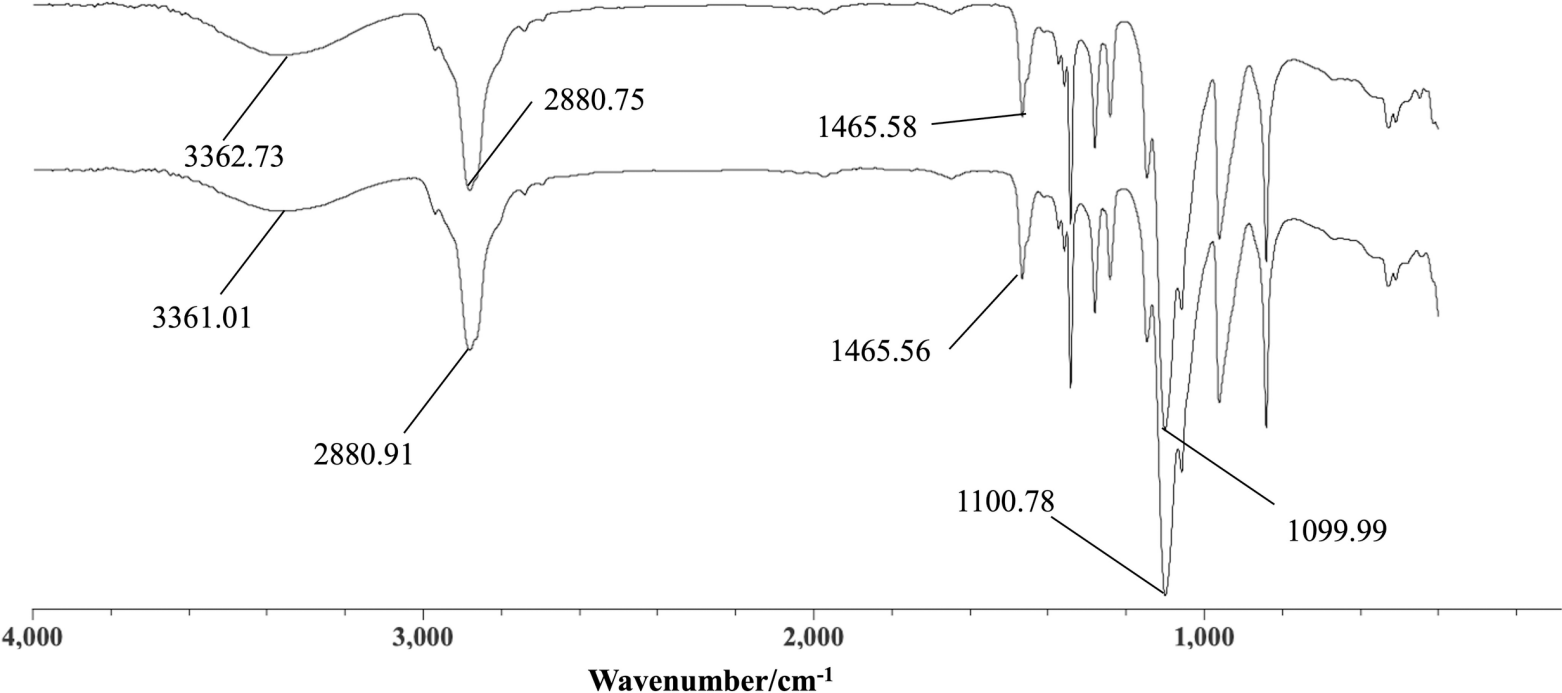
FTIR spectra of (1) the Dha A@TSG and (2) the blank temperature-sensitive gel. Abbreviations: FTIR, Fourier-transform infrared.

In the FTIR spectrum of poloxamer, the broad peak at 3300–3500 cm^-1^ resulted from the stretching vibration of hydroxyl groups (O-H); the peak at 3000–2843 cm^-1^ appeared from the antisymmetric stretching vibration of carbon-hydrogen bonds (C-H); the peak at 1146 cm resulted from the bending vibration of C-O, and the peak at 1300–1500 cm^-1^ appeared from the deformation vibration of C-H. The FTIR spectra of the Dha A@TSG and the blank gel were similar (Fig 1), indicating that the mixing of Dha A and gel molecules does not form covalent bonds, but that this mixing instead occurs through only intermolecular forces, such as hydrogen bonds.

### 2.3. Scanning electron microscopy

**Fig 2:**
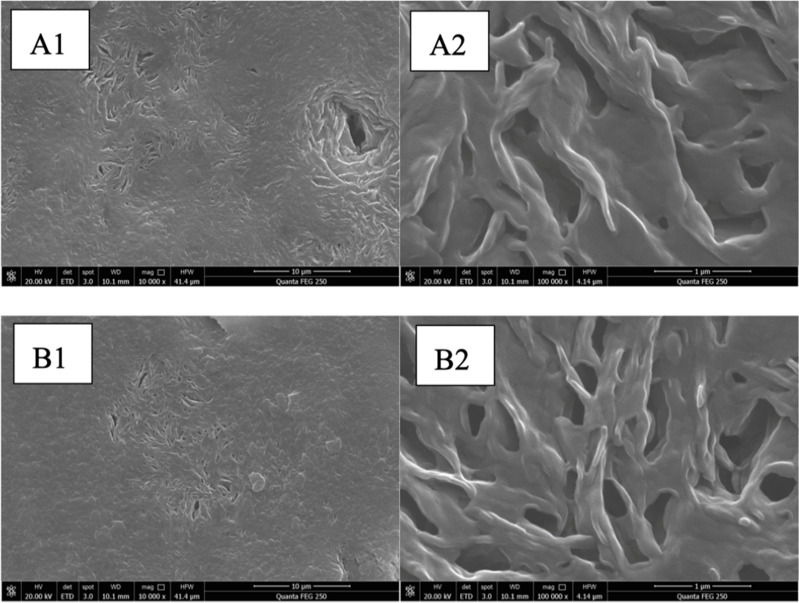
Scanning electron micrographs of the Dha A@TSG (A1, A2) and the blank temperature-sensitive gel(B1, B2). The magnifications for A1 and A2 were 10K, while those for B1 and B2 were 100K.

Scanning electron microscopy revealed that the surfaces of the Dha A@TSG and the blank gel consisted of rough and reticulated porous structures as shown in Fig 2. The reticulated structure morphology and pore size of both samples were similar, suggesting that the addition of Dha A did not significantly affect the surface morphology of the temperature-sensitive gel.

### 2.4. Rheological analysis

**Fig 3:**
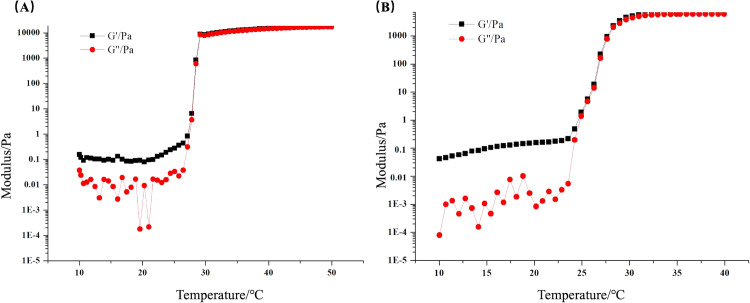
Temperature dependence curves of the energy storage modulus (G′) and loss modulus (G″) of (a) Dha A@TSG and (b) the blank temperature-sensitive gel.

It can be seen from Fig 3A that the energy storage modulus and loss modulus of the Dha A@TSG did not change significantly when the temperature ranged between 10°C and 24°C. When the temperature was above 25°C, the energy storage modulus and loss modulus of the Dha A-loaded temperature-sensitive gel started to increase rapidly; however, the energy storage modulus was smaller than the loss modulus; thus, the system existed in a liquid state. When the temperature was about 27°C, the energy storage modulus and loss modulus of the Dha A@TSG became comparable, and the liquid gel gradually transitioned to a semi-solid state. The temperature dependence curves of the energy storage modulus and loss modulus of the blank temperature-sensitive gel are shown in Fig 3B. Notably, the gelation temperature of the blank temperature-sensitive gel was about 26°C, indicating that the addition of Dha A did not significantly change the gelation temperature.

**Fig 4:**
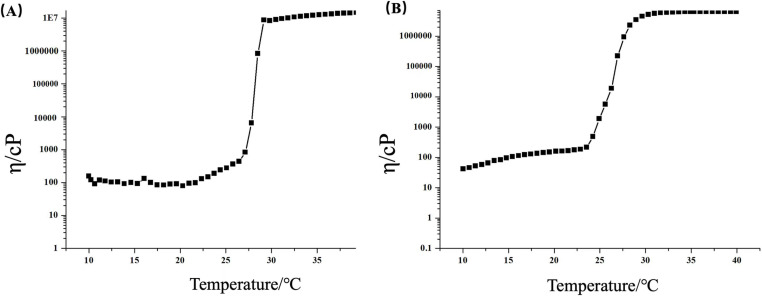
Temperature dependence of the viscosity of (a) Dha A@TSG and (b) the blank temperature-sensitive gel.

The viscosity of the Dha A@TSG did not change significantly with the increase of temperature from 10°C to 25°C (Fig 4A). However, when the temperature was raised to 28°C, the viscosity of the Dha A@TSG increased rapidly to 10^7^ cP. The liquid gel was gradually transformed into a semi-solid state, and its gelation temperature was between 25°C and 28°C. As shown in Fig 4B, the maximum viscosity of the Dha A@TSG also reached 10^7^ cP, and its gelation temperature was between 24°C and 28°C.

### 2.5. Dha A enzyme activity analysis

**Fig 5:**
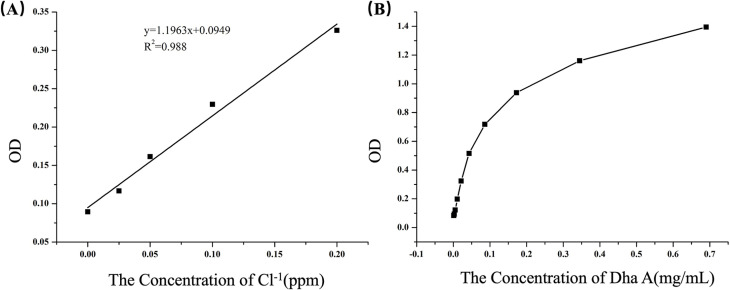
Determination of Dha A enzyme activity by the chloride ion assay method: (a) Relationship between chloride ion concentration and absorbance. (b) Relationship between absorbance and Dha A enzyme concentration.

As shown in Fig 5A, a linear relationship was maintained between chloride ion concentration and absorbance when the chloride ion concentration was between 0 and 0.2 ppm. This indicated that the chloride ion detection method could be used to determine of trace amounts of chloride ions produced by the Dha A enzymatic digestion of the mustard simulant bis(2-chloroethyl) ether. It was noticeable from Fig 5B that the absorbance gradually stabilized as the enzyme concentration in the reaction system increased, with the substrate concentration kept constant. According to the requirement of photometric errors, the measured absorbance was in the range of 0.2–0.8. The original concentration of the Dha A solution was 0.69 mg/mL, while the enzyme concentration in both free Dha A and the Dha A@TSG was 0.086 mg/mL (Fig 5B).

### 2.6. Catalytic efficiency analysis of Dha A

**Fig 6:**
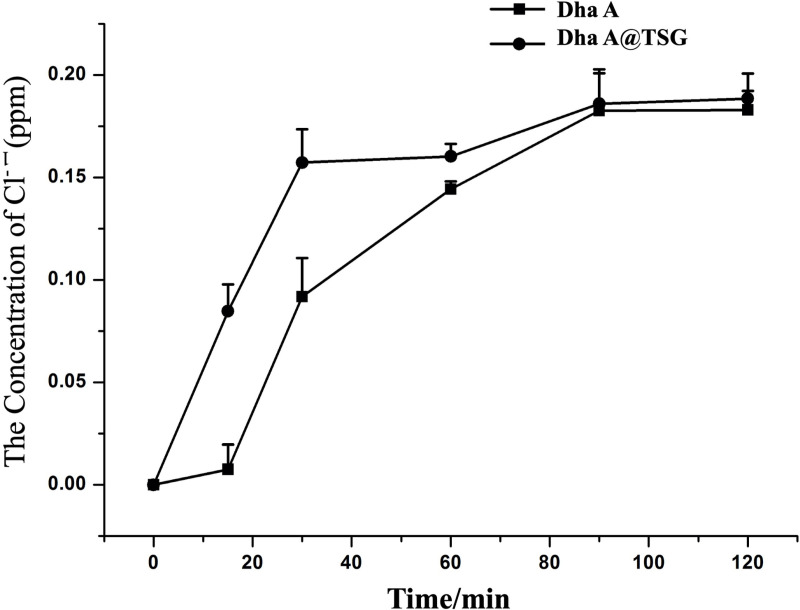
Chloride ion generation curves resulting from the catalytic degradation of bis(2-chloroethyl) ether by free Dha A and the Dha A@TSG.

Fig 6 showed the chloride ion generation curves resulting from the catalytic degradation of the mustard simulant bis(2-chloroethyl) ether by free Dha A and the Dha A@TSG at the human body surface temperature (32°C). It was observable that at 15 min and 30 min, the concentration of chloride ions generated from the degradation of bis(2-chloroethyl) ether by Dha A in the gel was significantly higher than that generated by free Dha A. The amount of chloride ions generated in the system gradually reached the equilibrium after 60 min.

### 2.7. Dha A enzymatic kinetic assay

**Fig 7:**
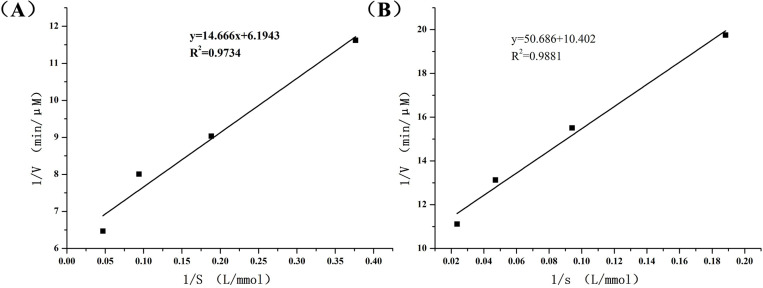
(a) Lineweaver-Burk curves of the free Dha A-degraded substrate. (b) Lineweaver-Burk curves of the Dha A@TSG -degraded substrate.

As poloxamer was a nonionic surfactant (Table 2), it increased the affinity of bis(2-chloroethyl) ether to Dha A, thus improving the catalytic efficiency of the enzyme (Fig 7).

**Table 2:** Enzymatic kinetic parameters.

	Km (µmol/L)	Kcat (s^−1^)	Kcat/Km
Free Dha A	5.43	1.14	0.21
Dha A@TSG	2.37	1.87	0.79

### 2.8. Stability test

**Fig 8:**
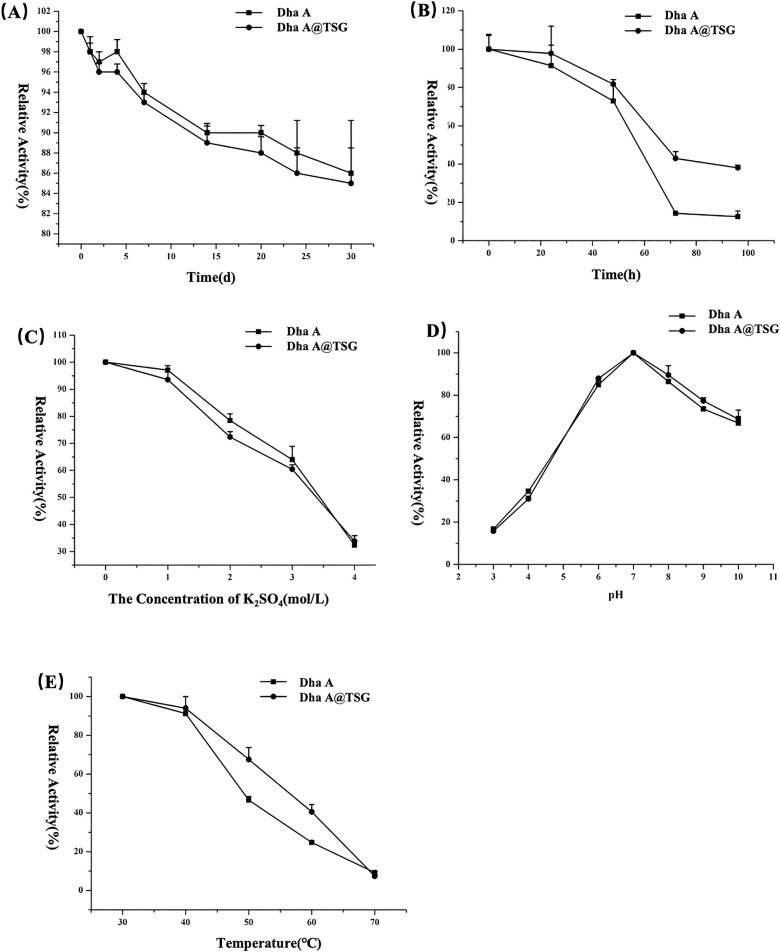
Stability of Dha A loaded on the poloxamer temperature-sensitive gel: (a) storage stability at 4°C, (b) storage stability at 32°C, (c) stability at high salt concentrations, (d) pH stability, and (e) thermal stability.

It was noticeable from Fig 8A that the residual activity of Dha A in both the free Dha A solution and the Dha A-loaded temperature-sensitive gel was greater than 85% in the first 30 days. However, the residual activity of Dha A in the Dha A-loaded temperature-sensitive gel was not significantly higher than that in the free Dha A solution. It was evident from Fig 8B that the residual activity of Dha A in the Dha A-loaded gel was higher than that in the free Dha A solution throughout the testing phase at 32°C. The residual activity of Dha A in the free Dha A solution was significantly low after 72 h, whereas the residual activity of Dha A in the Dha A-loaded temperature-sensitive gel was still about 38% after 96 h. Fig 8C revealed that the residual activity of Dha A in the Dha A@TSG and the free Dha A solution decreased gradually with increasing potassium sulfate solution concentration, and no significant difference in the residual activity of Dha A in these two systems was observed. Fig 8D showed that the activity of Dha A in both the Dha A-loaded temperature-sensitive gel and the free Dha A solution reached a maximum at pH =  7, and the optimal pH of Dha A did not change in the poloxamer temperature-sensitive gel. The residual activity of Dha A in both systems decreased gradually. It was observable from Fig 8E that the residual activities of Dha A in the two systems were comparable after incubation in the water bath at 30°C and 40°C for 1 h. However, with increasing incubation temperature, the residual activity of Dha A in the Dha A@TSG was significantly higher than that in the free Dha A solution after incubation in the water bath at 50°C and 60°C for 1 h. In contrast, after incubation in the water bath at 70°C for 1 h, Dha A in both systems became inactive.

## 3. Discussion

The FTIR spectroscopy results revealed that Dha A did not chemically react with the poloxamer temperature-sensitive gel to form covalent bonds; instead, it was physically co-bonded with the gel through hydrogen bonding.

Poloxamer is an ABA-type terpolymer consisting of hydrophilic polyoxyethylene and lipophilic polyoxypropylene groups [37]. Dha A was rich in amino and hydroxyl groups. When the Dha A solution was added to a poloxamer thermosensitive gel system at 4°C, the amino groups in the protein molecules formed hydrogen bonds with hydroxyl groups in the poloxamer molecules and water molecules [38]. The rheological test results indicated that the gelation temperature of the poloxamer temperature-sensitive gel was consistent with the value measured by the inverted test tube method. The addition of Dha A did not affect the gelation temperature of the poloxamer gel because the small amount of Dha A could not change the gelling characteristics of the temperature-sensitive gel. When its temperature was lower than 25°C, the liquid poloxamer temperature-sensitive gel existed in a semi-solid state, and when the temperature was greater than 32°C, it transformed into the liquid state [39]. The gelation temperature of the Dha A@TSG was 25°C–28°C; thus, the poloxamer temperature-sensitive gel remained in the liquid state at room temperature and quickly transformed into a semi-solid state upon contact with human skin.

When a small amount of the Dha A@TSG was applied to human skin, it rapidly transformed into a semi-solid state within 30 s, and the viscosity reached 10^7^ cP.The enzymatic activity of Dha A was indirectly determined by measuring the concentration of chloride ions produced from the catalytic degradation of bis(2-chloroethyl) ether by Dha A. The trace amount of chloride ions was determined by a colorimetric reaction, and the colorimetric stability was not easily affected by the polarity and ionic strength of the test system. The poloxamer thermosensitive gel system could also accurately display color, and it had a good linear relationship between chloride ion concentration and absorbance [7].

The catalytic activity and stability of Dha A in the poloxamer gel system were investigated by comparing the enzymatic properties of Dha A in the Dha A@TSG and the free Dha A solution. The catalytic efficiency and enzymatic kinetics of Dha A in both the Dha A@TSG and the free Dha A solution were determined to investigate the effects of the main components of the poloxamer gel, such as poloxamer 407, glycerol, and poloxamer 188, on the enzymatic properties of Dha A. It was found that the components of the temperature-sensitive gel did not affect the catalytic activity of Dha A. As poloxamer as a nonionic surfactant and glycerol increases the solubility of bis(2-chloroethyl) ether, the affinity of Dha A with the substrate was improved, resulting in enhanced catalytic efficiency of Dha A [40]. The temperature of human skin is 32°C. Even areas of skin directly contaminated by mustard gas wound do not immediately heat up, and it might take 1 to 2 days after contamination for the body temperature to rise by 1 to 2°C [41]. Thus, 32°C was selected to measure the catalytic efficiency of Dha A in Dha A-loaded temperature-sensitive gel and the free Dha A solution. It was noticed that although the Dha A-loaded temperature-sensitive gel already existed in a semi-solid state at 32°C, the substrate was enzymatically dissolved quickly by the gel. Furthermore, the results revealed that, within 30 min, Dha A had higher catalytic efficiency in the temperature-sensitive gel than in the free Dha A solution.

It has been reported that long-term storage at high temperatures or heat treatments often lead to a significant reduction in the enzyme activity of Dha A, and this occurred because high temperatures destroyed the hydrated layers of Dha A and exposed the enzyme’s hydrophobic sites, causing irreversible aggregation, denaturation, and inactivation of enzymes [18,42,43]. The storage stability and thermal stability of Dha A in the poloxamer temperature-sensitive gel were improved at 32°C. The gelation temperature of the Dha A-loaded temperature-sensitive gel was about 25°C. When the system temperature was higher than 25°C, the poloxamer temperature-sensitive gel turned into a semi-solid state; thus, hydrophobic polyoxypropylene groups became dehydrated at this temperature, and then the affinity between the polyoxypropylene groups increases to form microspheres with the hydrophobic polyoxypropylene groups as the core and the hydrophilic polyoxyethylene groups as the shell; thus, the microspheres became entangled to form a tight three-dimensional structure [33, 44]. Dha A was dissolved in the poloxamer temperature-sensitive gel at 4°C. The poloxamer temperature-sensitive gel existed in a liquid state at 4°C; thus, poloxamer molecules were free, the molecular structure only contained ether bonds and a small number of hydroxyl groups, and the interaction between poloxamer and Dha A molecules was less. As the temperature increased, Dha A molecules were firmly fixed on the tight steric structure formed by poloxamer molecules; hence, the rigidity of Dha A increased, the entropic change of the enzyme was reduced, and the mutual aggregation of Dha A molecules was inhibited [45]. The storage stability of Dha A was not significantly improved by the poloxamer temperature-sensitive gel at 4°C, whereas its storage stability was partially improved at 32°C. Similarly, the thermal stability of the poloxamer temperature-sensitive gel increased at 50°C and 60°C probably for the same reason.

The catalytic active sites of Dha A were susceptible to protonation or were attacked by salt ions in a strong acid or high salt solution environment and became inactive [46,47]. Theoretically, the hydration layer formed on the Dha A surface could mitigate this protonation and salt ion attack and increase the pH and stability of Dha A in high salt solutions. However, during the experiment, under the addition of strong acids, strong alkalis, and salt solutions, the state of the poloxamer temperature-sensitive gel changed; thus, the stability of Dha A was not improved.

## 4. Materials and methods

### 4.1. Materials

Poloxamer 407 (P407) and poloxamer 188 (P188) were purchased from BASF, Ludwigshafen, Germany. Glycerol (Gly) was ordered from Sinopharm Chemical Reagent Co (Beijing, People’s Republic of China). All other reagents used in this experiment were of analytical grade. Dha A was synthesized and purified according to a method described in previous study [18].

### 4.2. Preparation of the poloxamer temperature-sensitive gel and the immobilization of Dha A

The synthesis and preparation of the temperature-sensitive gel of poloxamer was based on two studies [48,49]. The poloxamer temperature-sensitive gel was prepared through the following steps: A mixed solution of P407 (concentration =  18%, *wt*), P188 (concentration =  1%, *wt*), and glycerol (concentration =  10%, *wt*) was prepared and stored overnight in a refrigerator at 4°C to obtain poloxamer temperature-sensitive gel. The gel solution was then mixed with a Dha A protein solution (2.2 mg/mL) in an ice bath at a volume ratio of 30:1.

### 4.3. Determination of gelling properties

The gelling properties were determined by the inverted test tube method [50]. The blank poloxamer temperature-sensitive gel and the Dha A-loaded temperature-sensitive gel were removed from the refrigerator (4°C), and 3 mL of each gel was mixed in a glass test tube. The test tube was then placed in a water bath, and the temperature was gradually increased from 20°C to 50°C. During this process, the test tube was quickly inverted to observe the flow state of the gel. The temperature at which the temperature-sensitive gel changed from liquid to solid was defined as the gelation temperature. At this gelation temperature, the time required for the temperature-sensitive gel to solidify was termed the gelling time. Each experimental procedure was repeated three times.

### 4.4. Fourier-transform infrared (FTIR) spectroscopy

The infrared spectra of the samples were determined based on the potassium bromide (KBr) pressing method, with100mg of both the blank poloxamer temperature-sensitive gel lyophilized powder and the Dha A-loaded temperature-sensitive gel lyophilized powder.

### 4.5. Scanning electron microscopy

Lyophilized powders of the blank poloxamer temperature-sensitive gel and the Dha A-loaded temperature-sensitive gel were dried, pasted onto a double-sided conductive adhesive tape, sprayed with gold, and characterized using a high-resolution cold field-emission scanning electron microscope(SEM,SU8010,Hitachi,Japan).

### 4.6. Rheological analysis

The temperature-dependent viscoelastic properties of the Dha A-loaded temperature-sensitive gel and the blank temperature-sensitive gel were determined using an Anton Par MCR302 rotational rheometer and based on Balakrishnan’s method [51]. A 0.5 mL sample of each gel was placed between the two plates of the rheometer, and changes in its energy storage modulus (G’), loss modulus (G”), and viscosity with temperature (10–40°C) were measured at a frequency of 1 Hz and a strain of 0.1%. The temperature corresponding to the intersection of the energy storage modulus (G’) and the loss modulus (G”) was recorded as the gelation temperature of the temperature-sensitive gel.

### 4.7. Dha A enzyme activity analysis

The enzymatic activities of the gels were measured by a modiﬁed colorimetric assay [52]. Sodium chloride particles (100 mg) were weighed, dissolved in 1 L of deionized water, and then diluted to prepare different concentrations of sodium chloride solutions. Subsequently, 50 µ L of mercury thiocyanate solution and 100 µ L of ferric ammonium sulfate solution were added to 200 µ L sodium chloride solutions at varying concentrations, followed by shaking. Furthermore, 200 µ L of each solution was transferred into a 96-well plate, and their absorbance at 460 nm was measured. According to Iwasaki’s study, a range of chloride ion concentrations exhibiting a good linearity between absorbance and chloride ion concentration was selected [52]. Moreover, 200 µ L of free Dha A solutions with different enzyme concentrations were added to aqueous bis(2-chloroethyl) ether solution, and the reaction was carried out at 37°C for 1 h. The reaction was terminated by adding 50 µ L of 30% concentrated nitric acid to the resultant solutions. The chloride ion concentrations in the solutions were then calculated using the chloride ion detection method, and the optimal Dha A concentration was screened for subsequent enzymatic property analysis.

### 4.8. Catalytic efficiency analysis

Free Dha A solution and the Dha A@TSG, both with the same enzyme concentration were added separately to equal volumes of aqueous bis(2-chloroethyl) ether and incubated in a water bath at 32°C. The chloride ion concentrations in the systems were measured at 15, 30, 60, 90, and 120 min, according to the method described in the literature [52].

### 4.9. Dha A enzymatic kinetic assay

Equal volumes of the free Dha A solution and the Dha A-loaded temperature-sensitive gel, both containing the same enzyme concentration, were mixed separately with different concentrations of bis(2-chloroethyl) ether, and the chloride ion concentrations in the solutions were measured at 37°C for 20 min. The change in the chloride ion concentration of the substrate after natural hydrolysis for 20 min was used as a control, and kinetic equations were derived by plotting double inverse curves. The kinetic constants (Km and Vm) of Dha A for the substrate were then calculated from the plotted curves.

### 4.10. Stability test

The stability tests were performed following previously published methods, with some modiﬁcations [18]. To determine the residual activity of Dha A, equal amounts of the free Dha A solution and the Dha A-loaded temperature-sensitive gel, both with the same enzyme concentration were added separately to different concentrations of potassium sulfate solutions and incubated at room temperature for 1 h. Subsequently, different pH buffers were added to the resultant solutions, which were then kept at room temperature for another hour. Following this, the solutions were stored at 4°C and 32°C, respectively, before being placed in a water bath at 30°C, 40°C, 50°C, 60°C, and 70°C for 1 h. The residual activity of Dha A was determined according to the method described in Section 4.7.

## 5. Conclusions

This study explored the use of a poloxamer-based temperature-sensitive gel to immobilize Dha A, an enzyme that degrades mustard gas simulant. The gel improves Dha A’s stability and catalytic efficiency without altering its gelation properties. FTIR and SEM analyses confirmed the physical bonding of Dha A with the gel. Rheological tests showed that the gel remained liquid below 25°C and transitioned to a semi-solid state above 32°C, making it suitable for human skin application. Enzymatic assays revealed that Dha A exhibited higher catalytic efficiency in the gel, especially at 32°C, along with improved stability, retaining significant activity after 96h. These findings suggest that Dha A-loaded poloxamer gel holds promise as an effective skin decontaminant for mustard gas.
